# Synthesis of Seco-Chlorinated Derivatives of Phenanthroindolizidine Precursors via Friedel-Crafts Reaction

**DOI:** 10.3390/molecules15118501

**Published:** 2010-11-22

**Authors:** Songtao Li, Li Han, Jiang Liu, Yihan Hu, Dan Zheng, Yingbo Fu, Xueshi Huang

**Affiliations:** Department of Natural Products Chemistry, Pharmaceutical School, China Medical University, Shenyang 110001, China

**Keywords:** phenanthroindolizidine, antitumor, Friedel-Crafts reaction

## Abstract

In the course of synthesizing 3-demethyltylophorine (**1)** by Lewis acid catalyzed intramolecular Friedel-Crafts reaction starting from *N*-(3-hydroxy-2,6,7-trimethoxy-phenanthr-9-ylmethyl)-2-chloromethylpyrrolidine, two chlorinated phenanthrene derivatives *N*-(4,10-dichloro-3-hydroxy-2,6,7-trimethoxyphenanthr-9-ylmethyl)-2-chloromethylpyrrolidine (**4)** and *N*-(4-chloro-3-hydroxy-2,6,7-trimethoxyphenanthr-9-ylmethyl)-2-chloromethylpyrrolidine (**5)** were obtained. The structures of these compounds were determined by spectroscopic analysis.

## 1. Introduction

**Scheme 1:**
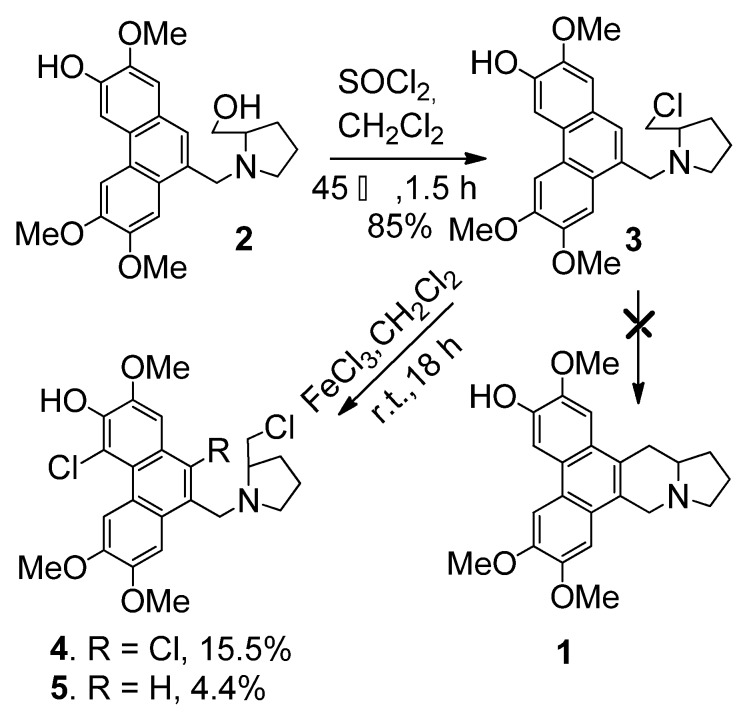
Synthesis of **4** and **5**.

Phenanthroindolizidine alkaloids, which were mainly isolated from Asclepiadaceae family [1], have attracted an intense attention because of their notable biological properties [2], especially the antitumor activity [3]. Many isolations, synthetic methodologies and biological activity studies [2,4] of this class of natural products have been reported since the first isolation of tylophorine in 1935 [5]. Despite of the potential biological activities, phenanthroindolizidine alkaloids have not yet been developed as clinical therapeutic drugs due to their serious central nervous system (CNS) side effects [1]. Inspired by this result, we attempt to synthesize a 3-demethyltylophorine (**1**, Scheme 1) and a series of derivatives based upon the functional 3-hydroxyl group on the phenanthrene ring, aimed at studying the impacts of the alternative polarities of these analogues on the antitumor activity and the CNS toxicity. 

The previous synthetic routes of phenanthroindolizidine alkaloids focused mainly on two directions: one was to finish the indolizidine unit followed by closing the phenanthrene ring [6,7,8,9]; the other involved the cyclization of phenanthrene firstly, then the synthesis of indolizidine ring system in the ultimate stage [10,11,12,13]. For the latter strategy, the typical method was via an amino (or amido) acid [14] to construct the indolizidine nucleus, and the commonly used building block was proline or its analogues [11]. In 1961, Govindachari and co-workers [15] reported that *N*-(2,3,6,7-tetramethoxy-phenanthrylmethyl)prolinol unsuccessfully linked to the phenanthrene ring under a variety of conditions attempted; In 1969, Chauncy *et al.* found that (–)-*N*-phenanthr-9-ylmethyl-L-prolyl bromide was failed to give the ring closure product by the Friedel-Crafts alkylation reaction, but polyphosphoric acid catalyzed acylation of the hydrochloride of (–)-*N*-phenanthr-9-ylmethyl-L-proline was successful [16]. However, the unstable ketone intermediate and low yield limited its application. In 1983, Rapoport *et al.* [11] attempted to catalyze the intramolecular Friedel-Crafts acylation of proline by Lewis acid, but it failed to give the ketone. In 2004, Banwell and Sydnes [17] reported that the seco-chlorinated derivatives of phenanthroindolizidine did not end up with the target ring closure products either. None of them, however, gave the detailed information of the reaction and the final products. As a part of our current work, we previously synthesized two intermediates: *N*-(3-hydroxy-2,6,7-trimethoxyphenanthr-9-ylmethyl)-L-prolinol (**2)** and *N*-(3-hydroxy-2,6,7-trimethoxyphenanthr-9-ylmethyl)-2-chloromethyl pyrrolidine (**3)**. On treatment with concentrated H_2_SO_4_/HOAc, **2** gave its acetate and hydrogen sulfate ester instead of the cyclized compound **1** [18]. Intramolecular Friedel-Crafts reaction of **3** catalyzed by chlorinated Lewis acids did not give **1** either (Scheme 1). In this paper, we reported the details of the products obtained from the intramolecular Friedel-Crafts reaction of **3**.

## 2. Results and Discussion

**Figure 1:**
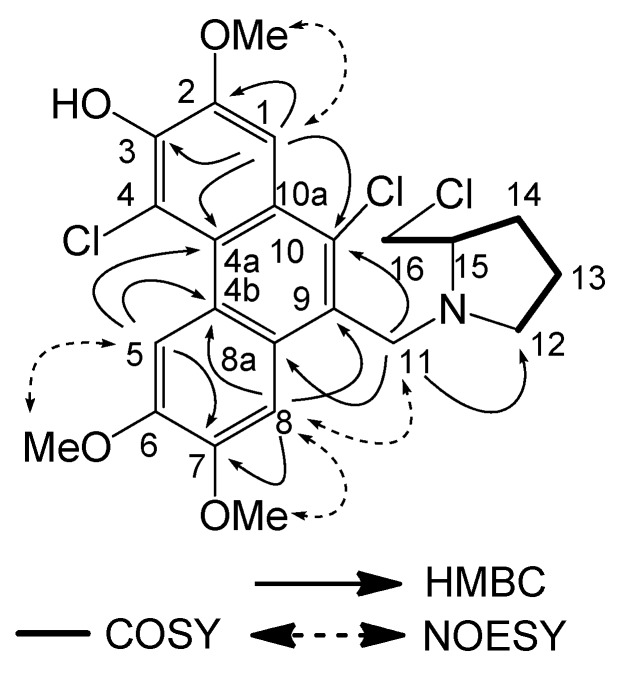
Relevant COSY, NOE and HMBC correlations of **4**.

Catalyzed by anhydrous AlCl_3_ in anhydrous CH_2_Cl_2_ at room temperature, **3** afforded a demethylated analogue **3a** (20%). The ^1^H NMR spectrum of **3a** was close similar to that of **3** except for the absence of signal due to a methoxyl group. Furthermore, the chemical shifts of aromatic protons at C-4 and C-5 positions (Δδ: 7.77-7.76 = 0.01 ppm) in **3a** were more approximate than those in **3** (Δδ: 7.86-7.82 = 0.04 ppm) [18]. Thus the structure of **3a** was established as 6-demethyl analogue of **3**. On treatment with anhydrous FeCl_3_ in anhydrous CH_2_Cl_2_ at room temperature, **3** furnished two chlorinated phenanthrene analogues **4** (15.5%) and **5** (4.4%). Reaction in anhydrous CS_2_, in the presence of anhydrous FeCl_3_, **3** also gave **4** (21.5%) as the main product (Scheme 1). In order to determine the structures of **4** and **5**, 1D NMR, 2D NMR and ESI mass spectra were determined for **4**. ESIMS indicated quasi-molecular ion peak at *m/z* 484 [M + H]^+^. The approximate relative intensities of the three peaks at *m/z* 484 [M + H]^+^, 486 [(M +2) + H]^+^ and 488 [(M + 4) + H]^+^ in a ratio of 3:3:1 in the ESI mass spectrum of **4** indicated the presence of three chlorine atoms in this structure and **4** was dichloro-substituted analogue of **3**. The molecular formula of C_23_H_24_Cl_3_NO_4_ was determined together with the analysis of its NMR data. There were only three aromatic proton signals at δ 9.20, 7.89 and 7.85 in the ^1^H NMR spectrum, which indicated another two chlorine atoms were located at phenanthrene ring. NOE effect observed in a NOESY spectrum between the proton at δ = 9.20 ppm and methoxyl group at δ = 4.08 ppm suggested that one chlorine atom could be located at C-4 position (Figure 1), together with lower field chemical shift of the proton at C-5 position caused by perieffect of the chlorine substituent at C-4 position. Another chloride group has to be located at C-10 position according to the NOE effects between the proton at δ = 7.89 ppm and methoxyl group at δ = 4.05 ppm and between the proton at δ = 7.85 ppm and methoxyl group at δ = 4.08 ppm. These evidences were supported by HMBC correlations as shown in Figure 1. All NMR data of **4** were assigned by COSY, NOESY, HMQC, and HMBC experiments (Figure 1). 

The approximate relative intensities of the three peaks at *m/z* 450 [M + H]^+^, 452 [(M + 2) + H]^+^ and 454 [(M + 4) + H]^+^ in a ratio of 9:6:1 in the ESI mass spectrum of **5** indicated the presence of two Cl atoms in this structure. Following the same logic used for compound **4**, the chlorine atom was determined to be located at C-4 position from lower field chemical shift at δ = 9.36 ppm for aromatic proton. The source of chlorination may come from the dissociative chlorine mixed in FeCl_3_. This result indicated that the electrophilicity of chlorine was stronger than primary chloroalkane in the electrophilic aromatic substitution reaction. C-4 and C-10 positions of compound **3** have higher chlorinated reactivities than other positions on the phenanthrene ring.

## 3. Experimental

### 3.1. General

All chemicals were commercially available and used without further purification unless otherwise stated. Anhydrous CH_2_Cl_2_ was dried over P_2_O_5_ and freshly distilled before use. Anhydrous CS_2_ was dried over anhydrous CaCl_2_ and freshly distilled before use. Melting points of the synthesized compounds were determined on a Digital Melting Point Appatatus XT4A and are uncorrected. 1D NMR spectra were recorded on Bruker ARX-300 spectrometer; 2D NMR spectra, on Bruker AV-600 spectrometer. ESIMS were recorded by Finnigan LCQ mass spectrometer. Column chromatography was carried out on silica gel (200-300 mesh, Qingdao Marine Chemical Ltd., Qingdao, China). Thin-layer chromatography (TLC) was performed on TLC silica gel 60 F254 plates (0.5 mm, Merck).

### 3.2. Synthesis of N-(3,6-dihydroxy-2,7-dimethoxyphenanthr-9-ylmethyl)-2-chloromethylpyrrolidine **(3a)**

To a suspension of anhydrous AlCl_3_ (150 mg, 1.12 mmol) in anhydrous CH_2_Cl_2_ (15 mL) was added a solution of **3** (50 mg, 0.12 mmol) in anhydrous CH_2_Cl_2_ (15 mL) under nitrogen. The reaction mixture was stirred for 24 h at room temperature and monitored by TLC. After the starting material was consumed, saturated Na_2_CO_3_ solution (20 mL) was added to the reaction system with vigorous stirring. The CH_2_Cl_2_ layer was separated and the aqueous layer was extracted with CH_2_Cl_2_ (20 mL × 2). The combined organic extracts were washed with brine, dried over anhydrous sodium sulfate, filtered, and concentrated. The crude product was purified by column chromatography on silica gel (CH_2_Cl_2_/P.E./ethyl acetate, 10:5:1 v/v) to afford **3a** (9.6 mg, 20%) as a pale brown solid; m.p. 206-208 °C; ^1^H NMR (300 MHz, CDCl_3_) δ: 1.62 (m, 1H, 0.5 × CH_2_), 1.75 (m, 2H overlapped, CH_2_), 2.12 (m, 1H, 0.5 × CH_2_), 2.20 (m, 1H, 0.5 × CH_2_N), 2.26 (m, 1H, 0.5 × CH_2_Cl), 2.90 (m, 1H, 0.5 × CH_2_N), 3.20 (m, 1H, 0.5 × CH_2_Cl), 3.80 (d, 1H, *J* = 12.9 Hz, 0.5 × ArCH_2_N), 3.93 (d, 1H, *J* = 12.9 Hz, 0.5 × ArCH_2_N), 4.02 (s, 6H, 2 × OCH_3_), 4.05 (m, 1H, CHN), 7.17 (s, 1H, Ar-H), 7.46 (s, 1H, Ar-H), 7.76 (s, 1H, Ar-H), 7.77 (s, 1H, Ar-H), 7.93 (s, 1H, Ar-H).

### 3.3. Synthesis of N-(4,10-dichloro-3-hydroxy-2,6,7-trimethoxyphenanthr-9-ylmethyl)-2-chloro methylpyrrolidine **(4)** and N-(4-chloro-3-hydroxy-2,6,7-trimethoxyphenanthr-9-ylmethyl)-2-chloromethylpyrrolidine **(5)**.

To a solution of **3** (100 mg, 0.24 mmol) in anhydrous CH_2_Cl_2_ (30 mL) was added anhydrous FeCl_3_ (0.4 g, 2.46 mmol) under nitrogen. The reaction mixture was stirred for 18 h at room temperature and monitored by TLC. After the starting material was consumed, 10% ammonia water (30 mL) was added to the reaction system with vigorous stirring. The CH_2_Cl_2_ layer was separated and the aqueous layer was extracted with CH_2_Cl_2_ (30 mL × 2). The combined organic extracts were washed with brine, dried over anhydrous sodium sulfate, filtered, and concentrated. The crude product was purified by column chromatography on silica gel (hexane/ethyl acetate, 3:1 v/v) to afford **4** (18 mg, 15.5%) as a white solid; m.p. 189-191 °C; ^1^H NMR (300 MHz, CDCl_3_) δ: 1.54 (m, 1H, 0.5 × 13-CH_2_), 1.66 (m, 1H, 0.5 × 14-CH_2_), 1.80 (m, 1H, 0.5 × 13-CH_2_), 2.09 (m, 1H, 0.5 × 14-CH_2_), 2.41 (m, 1H, 0.5 × 12-CH_2_), 2.58 (brd, *J* = 10.0 Hz, 1H, 0.5 × 16-CH_2_Cl), 2.73 (m, 1H, 0.5 × 12-CH_2_), 3.10 (brd, *J* = 10.0 Hz, 1H, 0.5 × 16-CH_2_Cl), 3.95 (m, 1H, 15-NCH), 4.05 (s, 3H, 7-OCH_3_), 4.08 (s, 6H, 2-OCH_3_ and 6-OCH_3_), 4.20 (d, *J* = 12.9 Hz, 1H, 0.5 × 11-CH_2_N), 4.22 (d, *J* = 12.9 Hz, 1H, 0.5 × 11-CH_2_N), 7.85(s, 1H, 1-ArH), 7.89 (s, 1H, 8-ArH), 9.20 (s, 1H, 5-ArH). ^13^C NMR (75 MHz, CDCl_3_) δ: 105.1 (C-1), 143.4 (C-2), 145.8 (C-3), 114.6 (C-4), 122.9 (C-4a), 123.3 (C-4b), 107.5 (C-5), 148.1 (C-6), 147.3 (C-7), 106.3 (C-8), 127.7 (C-8a), 127.7 (C-9), 129.2 (C-10), 124.1 (C-10a), 57.4 (C-11), 52.4 (C-12), 24.4 (C-13), 34.5 (C-14), 56.2 (C-15), 60.8 (C-16), 3 × OCH_3_ (56.2, 55.6, 55.3) and **5** (4.7 mg, 4.4%) as an off-white solid; m.p. 202-204 °C; ^1^H NMR (300 MHz, CDCl_3_) δ: 1.57 (m, 1H, 0.5 × CH_2_), 1.60 (m, 1H, 0.5 × CH_2_), 1.82 (m, 1H, 0.5 × CH_2_), 2.12 (m, 1H, 0.5 × CH_2_), 2.26 (m, 1H, 0.5 × CH_2_N), 2.41 (m, 1H, 0.5 × CH_2_Cl), 2.70 (m, 1H, 0.5 × CH_2_N), 3.06 (brd, *J* = 9.9 Hz, 1H, 0.5 × CH_2_Cl), 3.80 (d, *J* = 12.9 Hz, 1H, 0.5 × ArCH_2_N), 3.95 (d, *J* = 12.9 Hz, 1H, 0.5 × ArCH_2_N), 3.97 (brs, 1H, NCH), 4.05 (s, 3H, OCH_3_), 4.08 (s, 3H, OCH_3_), 4.10 (s, 3H, OCH_3_), 7.14 (s, 1H, Ar-H), 7.41 (s, 1H, Ar-H), 7.91 (s, 1H, Ar-H), 9.36 (s, 1H, Ar-H).

## 4. Conclusions

Intramolecular Friedel-Crafts reaction of **3** catalyzed by Lewis acids was infeasible under our conditions. Instead of giving 3-demethyltylophorine **(1)**, two chlorinated phenanthrene derivatives **4** and **5** were obtained and their structures were confirmed by spectroscopic analysis.
